# Point-of-care ClotPro thromboelastography to determine bleeding risk in two cats with factor XII deficiency

**DOI:** 10.1177/20551169251319138

**Published:** 2025-03-31

**Authors:** Vera Forer, Pavlos G Doulidis, Verena Steiner, Natali Bauer, Lisa Maria Kulmer, Nicole Luckschander-Zeller

**Affiliations:** 1 Clinical Center for Small Animals, Division of Small Animal Internal Medicine, Department for Small Animals and Horses, University of Veterinary Medicine, Vienna, Austria; 2 Tierklinik Sattledt, Traunkreis VET Clinic, Upper Austria, Austria; 3 Department of Veterinary Clinical Sciences, Clinical Pathology and Clinical Pathophysiology, Justus-Liebig-University Giessen, Giessen, Germany

**Keywords:** Coagulation, factor XII, thromboelastography, bleeding risk

## Abstract

**Case series summary:**

Factor XII (FXII) deficiency is a rare autosomal recessive genetic disorder in cats, leading to singular prolonged activated partial thromboplastin clotting time (aPTT) without increased bleeding risk. This case series describes two cats diagnosed with FXII deficiency using the ClotPro system, a point-of-care viscoelastic coagulation test. Both cats exhibited significantly prolonged aPTT and intrinsic pathway clotting time but maintained normal clot strength, as indicated by thromboelastography (TEG). FXII deficiency was confirmed through quantitative ELISA measurement. Despite prolonged clotting times, the cats did not demonstrate clinical bleeding, suggesting that FXII deficiency does not increase bleeding risk. However, larger controlled studies are necessary to further investigate the utility of TEG in cats with FXII deficiency or other coagulation disorders.

**Relevance and novel information:**

This study addresses the diagnostic challenges associated with FXII deficiency, a condition that can lead to misinterpretation of coagulation results and unnecessary treatments. It highlights the application of the ClotPro system in feline medicine, providing deeper insights into coagulation dynamics and confirming that FXII deficiency does not inherently increase bleeding risk despite prolonged clotting times.

## Introduction

Factor XII (FXII) deficiency is an autosomal recessive genetic disorder in cats caused by a mutation on the FXII gene.^1^ FXII initiates coagulation on artificial surfaces in vitro and leads to prolonged activated partial thromboplastin clotting time (aPTT) in affected cats, while prothrombin time (PT) usually remains within normal limits.^2^ In vivo coagulation is mainly activated through factor VII and tissue factor (TF), so that bleeding risk remains low in affected cats;^3^ however, FXII may be important in cardiovascular and inflammatory diseases.^4^ Prolonged coagulation times are often misinterpreted, leading to unnecessary administration of blood products or vitamin K1.^5^ Viscoelastic coagulation tests (VETs) are used for a deeper evaluation of the patient’s coagulation status and accurately determine the current bleeding risk, as different activators help to distinguish deficiencies of different pathways of the cascade.^6^ However, VETs suffer from pre-analytical susceptibility although their use might be beneficial as a point-of-care (POC) test;^6^ therefore, current implemented POC-VETs should be easy to handle and provide reliable results.^7^ In a previous study, we compared and described two methods using thromboelastographic variables for blood samples from healthy dogs;^8^ however, thromboelastographic data in cats are scarce.^9^ One case report found a hypocoagulable kaolin-activated thromboelastography (TEG) tracing and a concurrent normal TF-activated TEG tracing in two cats with confirmed FXII deficiency, but larger studies are lacking although crucial, to safely determine the actual bleeding risk in these patients can facilitate a better individualised treatment plan.^10^ Herewith, we plan to introduce the ClotPro system (Enicor, Haemonetics Corporation) as a novel POC-VET system in two cats with confirmed FXII deficiency.

## Case series description

### Case 1

**Figure 1:**
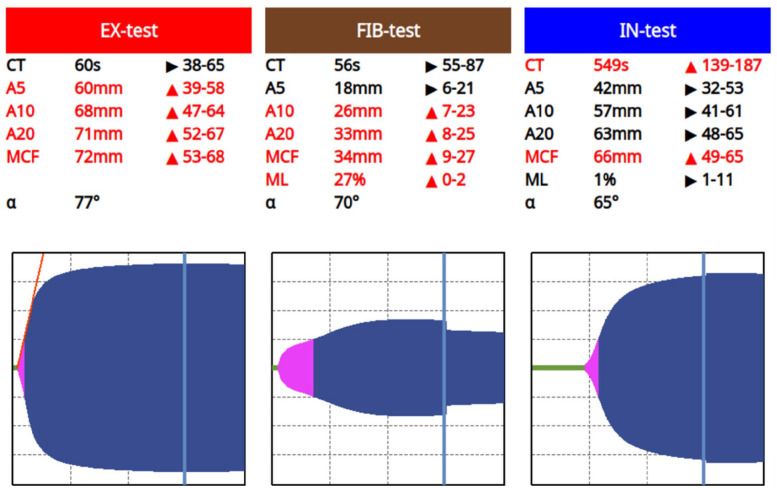
Figure illustrating the ClotPro thromboelastographic values for case 1. A5 = amplitude 5 mins after CT; A10 = amplitude 10 mins after CT; A20 = amplitude 20 mins after CT; CT = clotting time; MCF = maximum clot firmness; ML = maximum lysis

A 12-year-old spayed female shorthair outdoor cat was referred to the Small Animal Clinic of the University of Veterinary Medicine Vienna, Austria with acute onset of vomiting, inappetence and lethargy. A physical examination revealed mild dehydration, painful cranial abdomen and slightly icteric mucous membranes. The rest of the clinical examination was unremarkable. Abnormal laboratory findings included a mild total neutrophilia (16.76 10^3^/µl, reference interval [RI] 3.6–12.75), increased alanine transaminase (ALT) (1874 U/l, RI <100), increased gamma-glutamyl transferase (GGT) (8 U/l, RI <3.00), increased bilirubin (4.05 mg/dl, RI <0.20) and increased serum amyloid A (SAA) (12 mg/l, RI 0.80–1.60) (Table 1). Feline leukaemia virus (FeLV) antigen test and feline immunodeficiency virus (FIV) antibody test (SNAP FIV/FeLV Combo Test; IDEXX Laboratories) were negative. An abdominal ultrasound revealed a concernment in the papilla duodeni, compact sludge in the bile duct, as well as secondary cholestasis and congestion of the pancreatic duct. Treatment included the following intravenous fluids: methadone (0.2 mg/kg IV q4h); maropitant (1 mg/kg IV q24h); amoxicillin–clavulanic acid (20 mg/kg IV q8h); and omeprazole (1 mg/kg IV q24h). Based on these findings, a cholecystectomy was elected. One day preoperatively, coagulation panel testing was performed to evaluate bleeding risk. PT was within the normal range at 10 s (RI 8.0–10.0), aPTT was severely increased at >60 s (RI 8–17) and the thrombocyte count was 177 10^3^/µl (RI 180–430) (Table 2). As a result of the severely increased aPTT, a ClotPro TEG was performed. Upon thromboelastographic evaluation of the extrinsic pathway (EX), the clotting time (CT) was 60 s (RI 38–65), the amplitude 5 mins after CT (A5) was 60 mm (RI 39–58), the 10 min amplitude (A10) was 68 mm (RI 47–64), the 20 min amplitude (A20) was 71 mm (RI 52–67), the maximum clot firmness (MCF) was 72 mm (RI 53–68) and the maximum lysis (ML) was 2% (RI 2–12). Regarding the intrinsic system (IN), the CT was measured at 549 s (RI 139–187), A5 was 42 mm (RI 32–53), A10 was 57 mm (RI 41–61), A20 was 63 mm (RI 48–65), MCF was 66 mm (RI 49–65) and ML was 1%. Regarding the functional qualitative evaluation of fibrinogen level (FIB test), the CT was 56 s (RI 55–87), the A5 was 18 mm (RI 6–21), the A10 was 26 mm (RI 7–23), the A20 was 33 mm (RI 8–25), the MCF was 34 mm (RI 9–27) and the ML was 27% (RI 0–2) (Figure 1). Therefore, to confirm the suspicion of FXII deficiency, a quantitative measurement of FXII activity was performed. Two measurements were performed using the ELISA method, which measured FXII activity at 4% and 5%, respectively (RI > 50), confirming FXII deficiency. No complications during surgery were reported, including no excessive blood loss. After 3 days of hospitalisation, TEG was repeated. As values remained unchanged compared with the initial TEG, the cat was discharged and remained clinically stable until the last recheck 1 year later.

**Table 1:** The most relevant clinicopathological changes for case 1

Parameter	Value	Reference interval	
Total neutrophil count (10^3^/µl)	16.76	3.6–12.75	↑
Alanine aminotransferase (U/l)	1874	<100	↑
Gamma-glutamyl transferase (U/l)	8	<3	↑
Bilirubin (mg/dl)	4.05	<0.20	↑
Serum amyloid A(mg/l)	12	0.80–1.60	↑

Arrows indicate increased value

**Table 2:** Initial values of the coagulation parameters for case 1

Parameter	Value	Reference interval	
Thrombocytes (10^3^/µl)	177	180–430	–
Prothrombin time (s)	10	8–10	–
Activated partial thromboplastin clotting time (s)	>60	8–17	↑
Thrombin time (s)	14	<22	–

Arrow indicates increased value

### Case 2

**Figure 2:**
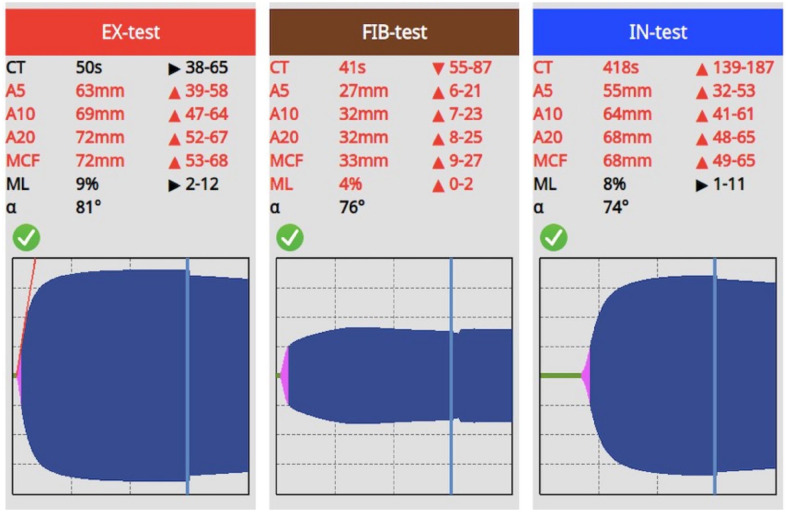
The ClotPro thromboelastographic values for case 2. A5 = amplitude 5 min after CT; A10 = amplitude 10 min after CT; A20 = amplitude 20 min after CT; CT = clotting time; MCF = maximum clot firmness; ML = maximum lysis

A 2-year-old male intact Maine Coon cat was referred to the clinic with a history of recurrent lethargy, anorexia and high rectal temperature (39.4°C). The referring veterinarian suspected feline infectious peritonitis (FIP). Intravenous fluids and antibiotic therapy were initiated by the referring veterinarian because of the fever (amoxicillin-clavulanic acid 20 mg/kg IV q8h). Bloodwork revealed lymphopenia (680/µl, RI 900–5100), decreased blood urea nitrogen (18 mg/dl, RI 20–65), slightly decreased total protein (5.59 mg/dl, RI 6–7.5) and alkaline phosphatase (64 U/l, RI <30). Elevated potassium (5.7 mmol/l, RI 3.5–5), phosphate (1.77 mmol/l, RI 0.8–1.6) and increased SAA (167 mg/l, RI <5) were also noted (Table 3). FeLV antigen and FIV antibody tests (SNAP FIV/FeLV Combo Test; IDEXX Laboratories) were negative. A direct Coombs test was negative and the thoracic radiographs were unremarkable. Abdominal ultrasound indicated generalised abdominal lymphadenopathy, hepatomegaly and splenomegaly. Based on the ultrasound findings, fine-needle aspiration (FNA) of the liver, spleen and the abdominal lymph nodes was performed. Cytological and molecular (real-time PCR) examinations of the aspirated material confirmed the diagnosis of FIP. Before FNA, coagulation testing was performed. aPTT was severely increased (50 s, RI 8–17), while PT and TT remained within normal range (10 s, RI 8–10 and 14.6 s, RI <20, respectively) (Table 4). Upon thromboelastographic evaluation of the EX, the CT was 50 s (RI 38–65), the A5 amplitude was 63 mm (RI 39–58), the A10 was 69 mm (RI 47–64), the A20 was 72 mm (RI 52–67), the MCF was 72 mm (RI 53–68) and the ML was 9% (RI 2–12). Regarding the IN test, the CT was 418 s (RI 139–187), the A5 was 55 mm (RI 32–53), the A10 was 64 mm (RI 41–61), the A20 was 68 mm (RI 48–65), the MCF was 68 mm (RI 49–65) and the ML was 8% (Figure 2). Regarding the FIB test, the CT was 41 s (RI 55–87 s), the A5 was 27 mm (RI 6–21), the A10 was 32 mm (RI 7–23), the A20 was 32 mm (RI 8–25), the MCF was 33 mm (RI 9–27) and the ML was 4% (RI 0–2) (Figure 2). A quantitative ELISA (same as in case 1) measurement of FXII activity was performed to confirm the suspicion of FXII deficiency. The FXII measurement twice showed a value of 10%. After 9 days, the cat was discharged.

**Table 3:** The most relevant clinicopathological changes for case 2

Parameter	Value	Reference interval	
Lymphocyte count (/µl)	680	900–5100	↓
Blood urea nitrogen (mg/dl)	18	20–65	↓
Total protein (mg/dl)	5.59	6–7.5	↓
Alkaline phosphatase (U/l)	64	<30	↑
Potassium (mmol/l)	5.7	3.5–5	↑
Phosphate (mmol/l)	1.77	0.8–1.6	↑
Serum amyloid A (mg/l)	167	<5	↑

Arrows indicate increased value

**Table 4:** Initial values of the coagulation parameters for case 2

Parameter	Value	Reference interval	
Thrombocytes (10^3^/µl)	128	180–430	–
Prothrombin time (s)	10	8–10	–
Activated partial thromboplastin clotting time (s)	50	8–17	↑
Thrombin time (s)	14.6	<20	–

Arrow indicates increased value

## Discussion

FXII deficiency is a relatively uncommon genetic condition found in cats, often identified incidentally during routine blood coagulation testing.^11^ This condition typically does not result in clinical bleeding, meaning that affected cats generally do not exhibit signs of excessive bleeding or bruising, despite the deficiency.^8^ The current literature estimates that the prevalence of moderate to severe FXII deficiency in domestic cats is approximately 2% of the general feline population.^1^ In contrast to cats, FXII deficiency appears to be even less commonly reported in dogs, with only a handful of case reports documented in the literature.^1^ This suggests that while the condition may exist in dogs, it has not been observed frequently enough to draw firm conclusions about its prevalence or clinical significance in this species. In the two feline cases discussed in this report, both cats presented with normal PT. However, they had a prolonged clotting time in the IN test of the TEG and a prolonged aPTT, where FXII plays a key role. Despite this abnormal finding, both cats demonstrated normal clot strength, as measured by the specific tests assessing the mechanical properties of the clot. This finding is crucial as it confirms that, despite the prolonged clotting times, the cats did not have an increased risk of bleeding in practical, clinical terms.

The diagnosis of FXII deficiency in these cases was definitively confirmed through quantitative measurement of FXII levels in the blood. This method provides a precise determination of the extent of the deficiency, ensuring that the prolonged IN-PTT was indeed due to low FXII levels and not due to other factors. This detailed analysis underscores the importance of comprehensive coagulation testing in diagnosing FXII deficiency, as well as the need to interpret these results in the context of the overall clotting function, which, as shown in these cases, may remain intact despite the deficiency. In this case report, several limitations should be considered. First, the absence of an established RI for cats necessitated the use of parameters derived from human medicine, which may not fully reflect feline physiology. In addition, as this is a report of two cases, no reliable conclusion regarding bleeding risk can be made. Furthermore, both cats presented with disease that required hospitalisation, a fact that could potentially influence coagulation parameters. Lastly, the case series design inherently lacks control groups, which restricts the ability to establish causality or generalise findings.

## Conclusions

Both cats in this case series demonstrated normal clot strength and amplitude despite a prolonged clotting time in the intrinsic pathway and aPTT prolongation, suggesting that FXII deficiency does not necessarily correlate with an increased bleeding risk. However, to better evaluate the clinical significance of this finding and to establish more definitive guidelines, prospective controlled studies involving a larger number of cases are needed.
